# Patient Aggression and Burnout in Psychiatry Inpatient Staff: A Cross-Sectional Survey

**DOI:** 10.1192/bjo.2026.11636

**Published:** 2026-06-30

**Authors:** Mohamed Jalloh, Samukeliso Fundira, Vasudevan Krishnan

**Affiliations:** 1 St Georges Hospital, Midlands Partnership University NHS Foundation Trust, Stafford, United Kingdom; 2 Royal Stoke University Hospital, University Hospitals North Midlands, Stoke-On-Trent, United Kingdom

## Abstract

**Aims:**

Psychiatry inpatient staff are frequently exposed to patient aggression, including verbal abuse and physical threats. While aggression is widely recognised as a workplace hazard, the relative contribution of different aggression types to staff burnout remains poorly explored. Therefore, we aim to examine the association between patient aggression exposure and burnout among psychiatry inpatient staff.

**Methods:**

We conducted an anonymous cross-sectional survey of inpatient psychiatry staff (n=115) across multiple ward types. Burnout was measured using a 7-item composite scale (Cronbach’s α;=0.915), which was approximately normally distributed (Shapiro–Wilk p=0.147). Exposure to patient aggression over the preceding six weeks was assessed using frequency-based Likert items covering verbal aggression, threats of harm, attempted assault, physical assault, sexual harassment, and racist or discriminatory abuse. Analyses included Pearson correlation, one way ANOVA to assess dose response relationships, and multivariable linear regression adjusting for professional role group, years working in psychiatry, and work pattern. All statistical analyses were performed using IBM SPSS Statistics.

**Results:**

Mean burnout score was 1.92 (SD 0.87). Verbal aggression was reported by 93.9% of respondents, with 41.8% experiencing it weekly or more often. Other aggression types were also common, including threats of physical harm (73.9%) and physical assault (58.3%).In unadjusted analyses, burnout was positively correlated with verbal aggression frequency (r=0.21, p=0.024), but not with other aggression types. Mean burnout differed significantly across verbal aggression frequency categories (F (5,109)=2.43, p=0.040), demonstrating a dose response pattern. In multivariable regression, verbal aggression remained independently associated with higher burnout scores (B=0.16, 95% CI 0.03–0.29; p=0.014).

**Conclusion:**

Among psychiatry inpatient staff, frequent verbal aggression is common and shows a dose response association with burnout. These findings highlight verbal aggression as a key occupational stressor and a potential target for prevention strategies in inpatient psychiatric settings.

